# Structured hysteroscopic examination of uterine niches: a modified Delphi procedure

**DOI:** 10.52054/FVVO.16.3.036

**Published:** 2024-09-30

**Authors:** N Min, R.A. de Leeuw, L.F. van der Voet, A Di Spiezio Sardo, P.N. Barri-Soldevila, M Dueholm, O Donnez, E Saridogan, T.J. Clark, H.A.M. Brolmann, A.L. Thurkow, D Jurkovic, T van den Bosch, T Bourne, W.J.K. Hehenkamp, J.A.F. Huirne

**Affiliations:** Amsterdam University Medical Center, location Vrije Universiteit Amsterdam, Department of Obstetrics & Gynaecology, De Boelelaan 1117, Amsterdam, The Netherlands; Amsterdam Reproduction and Development research institute, Amsterdam, The Netherlands; Deventer Ziekenhuis, Obstetrics and Gynaecology, Deventer, The Netherlands; Università degli Studi di Napoli “Federico II”, Obstetrics and Gynaecology, Napoli, Italy; Institut Universitari Dexeus- Càtedra d’Investigació en Ginecologia-, Departament d’Obstetricia i Ginecologia-, Barcelona, Spain; Aarhus University, Department of Clinical Medicine - Department of Obstetrics and Gynaecology, Aarhus, Denmark; Complex endometriosis center, Polyclinique Urbain V (Elsan group), Avignon, France; University College London Hospitals, Reproductive Medicine and Minimal Access Surgery Units, London, United Kingdom; Birmingham Women’s & Children’s Hospital, University of Birmingham, Birmingham, United Kingdom; University College Hospital, Gynaecology Diagnostic and Outpatient Treatment Unit, London, United Kingdom; Universitair Ziekenhuis Leuven, Department Gynaecology and Obstetrics, Leuven, Belgium; Queen Charlotte’s and Chelsea Hospital, Imperial College London, London, United Kingdom

**Keywords:** Delphi technique, caesarean scar defect, uterine niche, hysteroscopy, diagnostic imaging, classification

## Abstract

**Background:**

Uterine niches in the Caesarean section scar are seen in approximately half of women with a history of caesarean delivery. Whilst a structured ultrasound assessment of caesarean defects has been described, there is no consensus on a structured hysteroscopic evaluation.

**Objectives:**

To propose a methodology for a structured hysteroscopic evaluation of uterine niches.

**Materials and Methods:**

We conducted a modified Delphi procedure, including two online rounds and two face-to-face meetings of the members of the ESGE Uterine Niches Working Group. The taskforce members have extensive experience in hysteroscopic niche evaluation. The consensus was predefined as a Rate of Agreement of at least 75%.

**Results:**

Thirteen experts participated in this modified Delphi procedure. There was consensus on the need for a standardised methodology and the hysteroscopic definition of a niche as any indentation in the myometrium at the site of a previous CS. There was consensus that a hysteroscopic evaluation of a niche must be combined with ultrasound to measure the residual myometrial thickness. In addition, it was agreed that niches should be subclassified as ‘simple’, ‘simple with one branch’, or ‘complex’. There was consensus that the following items should be described during a hysteroscopic niche evaluation: the number of niches, the size in relation to the size of cervical canal, the presence of polyps, crypts, cysts, fibrotic tissue, blood, mucus, placental remnants, a dynamic valve, the appearance of the endometrium, the number of blood vessels and bleeding from blood vessels within the defect.

**Conclusion:**

Using a modified Delphi procedure with international experts, consensus was achieved on the hysteroscopic evaluation and classification of niches in the uterine caesarean section scar.

**What is new?:**

A structured registration form was developed to aid consistency in hysteroscopic niche reporting.

## Introduction

With rising caesarean section (CS) rates in the last decades, there has been an increasing focus on long-term complications of a caesarean scar (World Health Organization Human Reproduction Programme, 2015; Betran et al., 2021). In approximately half of the women with a caesarean section, a scar defect, also called a niche, is visible in the uterus. The prevalence of a niche is estimated to be between 56 and 78% in women examined by ultrasound (BijdeVaate et al., 2014; BijdeVaate et al., 2011; Roberge et al., 2012; Antila-Langsjo et al., 2018; Klein Meuleman et al., 2023a) and between 31-100% in women evaluated by hysteroscopy (Borges et al., 2010; Fabres et al., 2003; El-Mazny et al., 2011; van der Voet et al., 2017; Tower and Frishman, 2013; Tulandi and Cohen, 2016; Klein Meuleman et al., 2023a).

For a niche evaluated by ultrasound the agreed definition is an indentation at the site of the CS scar with a depth of at least 2 mm (Jordans et al., 2019). The same authors described a systematic, structured measurement method of the niche based on a structured consensus derived from international experts. However, an equivalent structure and methodology has not been formulated for hysteroscopic niche evaluation. Different definitions have been proposed to describe a niche by hysteroscopy (Borges et al., 2010; van der Voet et al., 2017; Fabres et al., 2003) demonstrating the need to agree on a uniform definition and approach to evaluating and reporting a niche by hysteroscopy.

Niches in the uterine scar are related to abnormal uterine bleeding, dysmenorrhoea, and impaired fertility (Bij de Vaate et al., 2011; van der Voet et al., 2014; Tower and Frishman, 2013; Mashiach and Burke, 2021). A recently published article defined a disorder caused by a symptomatic niche, the Caesarean Scar Disorder (CSDi) (Klein Meuleman et al., 2023b). Different therapies have been developed to reduce niche-related symptoms. Laparoscopic or vaginal repair and hysteroscopic resection are effective treatments to reduce abnormal uterine bleeding and pain, as well as improving quality of life and reproductive outcomes in cases of secondary infertility (Mashiach and Burke, 2021; Donnez, 2020; Stegwee et al., 2020; Abdou and Ammar, 2018). A relationship between niche features and symptoms has yet to be fully elucidated although, BijdeVaate et al. (2011) showed that both the size of a niche expressed as the volume of the niche or as the ratio between the niche depth and the thickness of the adjacent wall as assessed by ultrasound are associated with postmenstrual spotting (van der Voet et al., 2014). But so far, there are no studies on the relationship between niche features assessed by hysteroscopy and niche-related symptoms. In addition, specific niche features that are prognostic for the success of niche therapy, including hysteroscopic resections, have not been studied.

In order to improve communication between clinicians and patients, and allow reliable comparisons between studies, a generally accepted definition of a niche and a systematic method of evaluation by hysteroscopy is needed. An accepted approach is a structured consensus method among international experts (Fink et al., 1984). We aimed to develop consensus on a standard hysteroscopic method to report and classify a niche by this consensus technique. Moreover, we aimed to determine relevant features that should be recorded to obtain an internationally accepted registration system that can be used in future studies.

## Method

**Figure 1:**
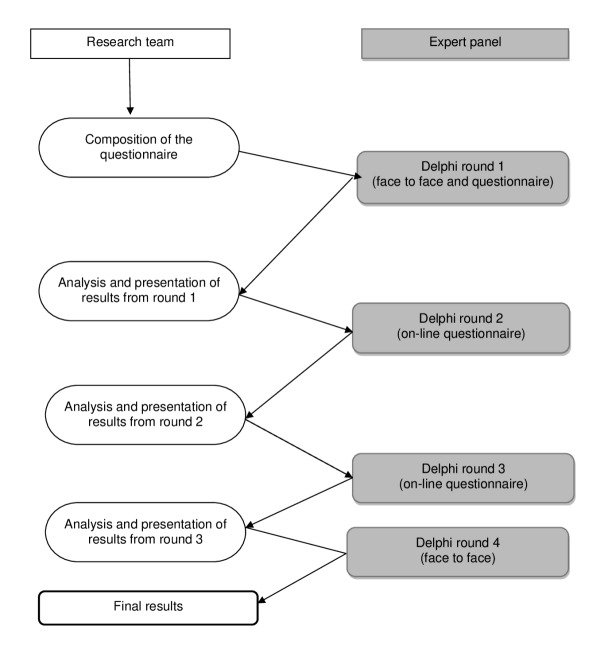
Study design. The modified Delphi method used in this study to define the definition of a niche evaluated with hysteroscopy and to develop a registration form.

This project was started as an ESGE Uterine Niches Working Group initiative. The Delphi procedure is a well-established method to reach consensus between international experts (Fink et al., 1984). This study used a modified version of the original Delphi method (see Figure 1). Within the Delphi process, the votes of all panel members are weighed equally. We continued until a consensus was reached, pre-defined as a Rate of Agreement (RoA) of 75% or greater, where RoA = (agreement – disagreement) / (agreement + disagreement + neutral) x 100% (Janssen et al., 2011; Jordans et al., 2022). The Delphi procedure started with a focus group of experts to determine which items should be discussed. This resulted in the first questionnaire, which was digitally sent to the Delphi panel. The expert panel was asked to comment on the questions and their answers after each subject. The results of each round, including the comments were reported anonymously to the participants in the next round. Reminder emails were sent to non-responders after seven days and after fourteen days.

### Expert panel recruitment

We selected our experts based on their membership of the international task force on niches of the European Society for Gynaecological Endoscopy (ESGE) or their recognised expertise in the hysteroscopic diagnosis and treatment of niches. They were considered experts when they performed at least 50 hysteroscopic evaluations and were actively involved in niche treatment and/or research.

### Literature search and focus group discussions

We started by performing a literature search on the hysteroscopic evaluation of niches. During a focus group meeting among Dutch experts, different videos of hysteroscopic evaluations of niches were discussed. The combination of the available literature and the focus group discussion led to a list of relevant items to discuss in the first round Delphi procedure. This first list was then evaluated by members of the ESGE Uterine Niches Working Group at the ESGE Annual Congress in Brussels in October 2016. During this meeting, a definition of a hysteroscopic niche and a list of potentially relevant items were formulated. The meeting was recorded and transcribed. All participants completed an anonymous written questionnaire and signed an informed consent.

### Modified Delphi procedure

After this first round, the digital Delphi procedure consisted of an online questionnaire organised via email. After confirmation of participation, the experts received an email containing a unique link to the online questionnaire. After seven, and after 14 days, a reminder email was sent to non-responders. Results were reported back anonymously until consensus was confirmed. Of all potential items, experts were asked whether they had to be included and how to define these items in open and closed questions. During the Delphi, responders could add outcomes that they considered important but were not provided in the first list. Non-responders of this round were not invited for the following rounds.

### Final consensus meeting

After three rounds, the final items without consensus were discussed during a meeting on June 28th, 2017, in Amsterdam. Here consensus was achieved by open discussion of these last items.

## Results

The Delphi procedure was performed between October 2016 and July 2017.

### Expert panel

We initially invited 21 experts for our Delphi procedure, nine experts could visit the initial face-to-face meeting, four could not join the meeting but were willing to participate in the online round, and eight experts did not respond. Therefore, 13 experts were included; after the first meeting, one expert was added based on the expertise in hysteroscopic niches, and one left without further participation.

The digital Delphi was sent to 13 experts, the response rate was 100%, and the last round had a response rate of 77%. Eight experts were able to join the final consensus meeting. All participants gave their final approval to the final results of the agreed items.

### Literature and focus group meeting results

The literature search provided 10 items. The focus group consisted of 8 Dutch gynaecologists, resulting in 13 potentially relevant items for the registration form and three additional questions about defining a niche. This list of 16 items was the start of the digital Delphi (see Appendix 1).

### Delphi results

During the first round of the Delphi, 24 additional questions were added, see Appendix 2. Consensus was achieved on 33 of the 40 items after two rounds. In the next round, two items were added. After the last round, consensus was achieved on 39 of the 42 items. Tables I-III show the consensus course per question. During the final agreement meeting, consensus was achieved on the last three items.

**Table I:** Features of the niche for hysteroscopic evaluation.

	First round	Second round	Third round	Final consensus meeting
Relevance				
The need for a standardised registration form for the evaluation of niches during hysteroscopy for both clinical and research purposes	89%			
What to record in the registration form				
Classification Use the following classification: simple niche, simple niche with one branch and complex niche^a^		85%		
Size Measurement of the depth and width of the niche in mm or cm is not useful since it can’t be done accurately during hysteroscopy. This has to be measured with ultrasound.	100%			
A subjective qualification of the size or volume of the niche is useful^a^		85%		
Use the subjective classification of size; small/medium/large/extreme^b^				100%
Describe If you can see the complete niche and the internal os in one view (1-2 cm below lower rim niche)^b^				100%
Localisation Describe the localisation of the niche: is it visible inside the cervical canal or more proximal inside the cavity?^a^		92%		
Define the position of the niche in the cervical canal by measuring the mm from the external os^a^		77%		
Features of the niche that should be registered^a^				
Presence of cystic formations (including ovula of Nabothi)		100%		
Presence of polyp-like structures		100%		
Presence of crypts		77%		
Presence of vessels		100%		
Presence of blood		100%		
Presence of mucus		100%		
Presence of fibrotic tissue		77%		
Presence of lateral branches		100%		
Presence of a dynamic valve in the niche (dynamic obstruction of the niche)		100%		
Presence of placental remnants		100%		
Presence of endometrium		69%	85%	
Number of niches		85%		
Description of all niches		85%		
Number of vessels (<5 or ≥5)		85%		
The fact if the vessel easily bleeds by releasing the pressure		69%	85%	
Features of the niche that should NOT be registered^a^				
The location of the niche in relation to the internal os		69%	85%	
The pattern of vessels		72%	85%	
The size of the vessels		38%	77%	

^a^ Item(s) added after the first round

^b^ Item added after the third round

**Table II:** Definitions of structural abnormalities.

	First round	Second round	Third round	Final consensus meeting
Definitions				
All agreed to use, when possible, the same definitions and terminology during ultrasound and during hysteroscopy	100%			
Definition of a Niche by hysteroscopy Any indentation in the myometrium at the site of a previous CS	89%	100%		
Definition of a branch A smaller part of the niche directing towards the serosa that has a smaller width then the niche itself^a^		85%		
Definition of a cystic formation Closed sac-like structure filled with fluid visible that is bulging the surface of the niche^a^		100%		
Definition of fibrotic tissue A focal hard tissue with a white surface without vessels^a^		92%		
Definition of a dynamic valve A dynamic partial or complete obstruction of the niche out-flow which moves when changing pressure^a^		85%		
Definition of a crypt Crypts are defined as cysts with an open connection to the niche^a^		77%		
Definition of a polyp Endometrium like focal growth^a^		31%	69%	100%

^a^ Item added after the first round

**Table III:** Performance of hysteroscopic evaluation.

	First round	Second round	Third round	Final consensus meeting
Advice on how to perform niche evaluation by hysteroscopya				
A hysteroscopic evaluation of a niche has to be combined with an ultrasound evaluation in order to measure the residual myometrium		100%		
Prevent dilatation during a diagnostic hysteroscopy in order to prevent changes of the niche appearance.		85%		
Record the diameter of the hysteroscope used		85%		
Preferably include a video or picture(s) are recorded in addition to the registration form		77%		
If a video / picture(s) are taken then start with an overview of the cervical canal and the niche		100%		
Store separate pictures of the abnormalities in the niche		77%		
Store an overview of the uterine cavity with tubal ostia		85%		
No need to take a video / picture(s) with both pressure and without pressure		38%	77%	

^a^ Items added after the first round

### Agreed recommendations and statements

**Figure 2:**
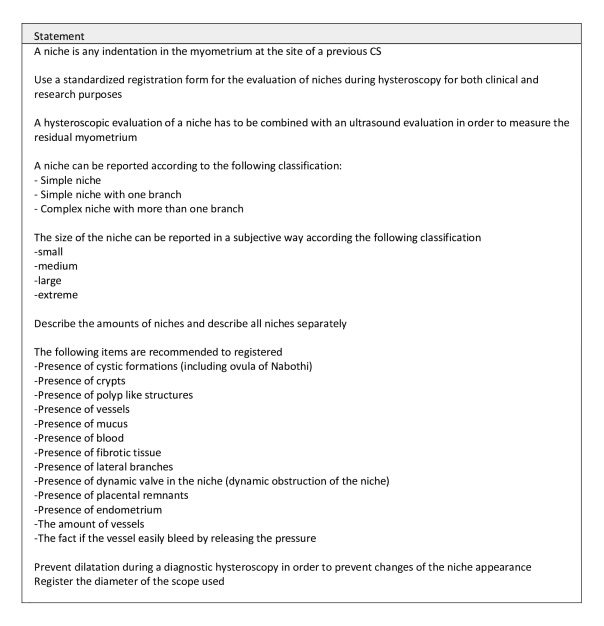
Final statements.

All final statements are summarised in Figure 2.

### Definition of a niche evaluated by hysteroscopy;

any indentation in the myometrium at the site of a previous CS (RoA 89%).

The experts agreed on the relevance of a structured evaluation (RoA 89%), but also that a hysteroscopic evaluation of a niche must be combined with an ultrasound evaluation to measure the thickness of the residual myometrium (RoA 100%). There was consensus on the fact that measuring the niche should be done by ultrasound (RoA 100%), however, a subjective qualification of the size or volume of the niche is useful (RoA 85%). This can be done by describing the niche as small, medium, large, or extreme in relation to the size of cervical canal (RoA 100%). There was consensus that describing the position of the niche in relation to the internal os was not valuable (RoA 85%), but the distance from the external os to the niche is useful (RoA 77%).

### Items to be structurally evaluated by hysteroscopy

**Figure 3:**
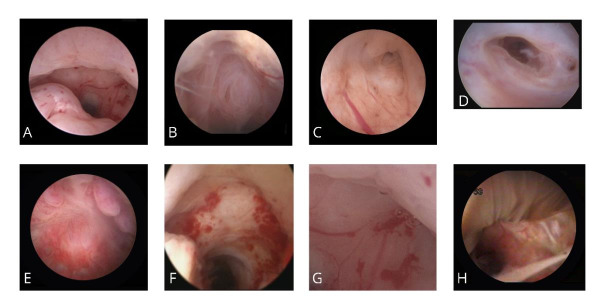
Items to be structurally evaluated by hysteroscopy: A. any indentation in the myometrium at the site of a previous caesarean section; B. cystic formations; C. crypts; D. lateral branches; E. polyp like structures; F. fibrotic tissue; G. abnormal vascular pattern; H. the presence of mucus.

It was agreed that these items should include: Cystic formations; Crypts; Lateral branches; Polyp like structures; Fibrotic tissue; Dynamic valve in the niche; Presence of blood, mucus, placental remnants, or endometrium; Vascularity i.e. the number of vessels and bleeding when distension pressure is released. These items are listed and defined in Table I and II and Figure 3. In addition to describing these items it was agreed that it is useful to add the estimated percentage of the niche that is filled with the structure in question.

Besides the items to be described, there was also agreement on how to perform the hysteroscopy (see Table III). The consensus was not to dilate before a diagnostic hysteroscopy (RoA 85%), and to record the diameter of the hysteroscope (RoA 85%).

**Figure 4:**
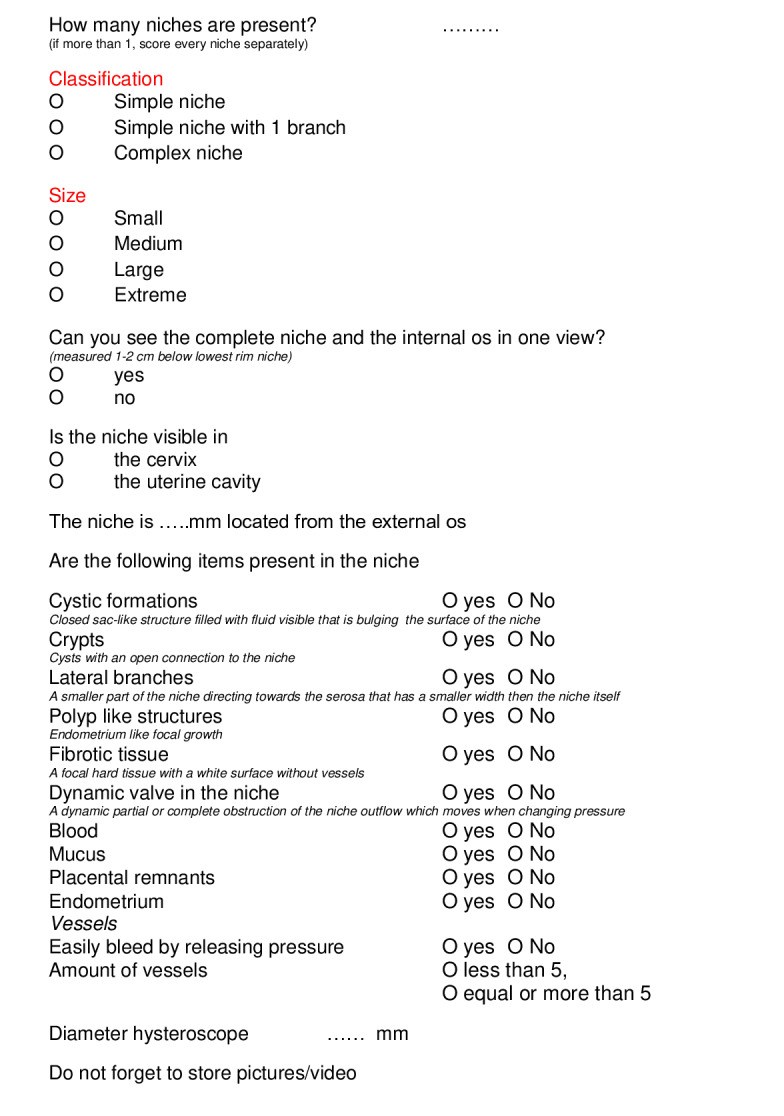
Hysteroscopic evaluation form uterine caesarean scar.

All these items and consensus statements are summarised in a record form, allowing a structured method of hysteroscopic evaluation (see Figure 4 and Appendix 3).

## Discussion

### Main findings

We performed a modified Delphi procedure on international experts resulting in a uniform classification of a niche and a structured list of items that should be evaluated and described during hysteroscopy. Additionally, there was complete consensus that a hysteroscopic evaluation of the niche must be combined with ultrasound to measure the thickness of the residual myometrium. Ultrasound seems to be the superior imaging modality to diagnose a niche (Klein Meuleman et al., 2023a). If a hysteroscopy is performed i.e. during surgery or because of inconclusive ultrasound, uniform recording is essential for future research and can potentially contribute to the development of future prognostic models.

### Strength and limitations

As far as we know, this modified Delphi procedure is the first to achieve consensus on a definition and classification of a niche evaluated by hysteroscopy. One of the strengths of our modified Delphi is that a diverse expert group was involved. Another strength is that we had both face-to-face focus groups and online Delphi rounds, allowing all experts the opportunity to discuss and add their experiences and new items. We developed a structured record form (see Figure 4) to be used in clinical practice and study settings.

A limitation of the current Delphi is the presence of only European experts. We could only attract experts from within Europe at the time of this Delphi. We are also aware that the Delphi rounds were in 2017, while this paper was prepared for publication in 2023. We believe, though, that the structured evaluation and pathology of the niche has stayed the same over the years. Another limitation is that the composed, structured form may include too many items. We are still determining which item will be relevant for clinical use or study purposes. The first step will be to evaluate the form in daily practice. We need to validate the form and study its performance in different populations.

Additionally, the inter- and intra-observer variation has yet to be studied. In order to increase the inter- observer agreement, we intend to standardise the hysteroscopic niche evaluation method. To identify and evaluate a niche in a diagnostic setting a diagnostic hysteroscopy performed without dilatation is most ideal, since dilatation may change the niche appearance. The diameter of the diagnostic hysteroscope can influence the outcome. Therefore, the diameter of the scope should be recorded. As discussed during this Delphi process, the use of digital images / videos to supplement written records was considered good practice. Furthermore, the need to evaluate the niche at hysteroscopy both on entry into the uterine cavity and also following uterine cavity distension and assessment was considered helpful from a technical point of view.

### Interpretation

Although several studies describe aspects of the niches, a relationship between hysteroscopic features and niche-related symptoms is not yet studied (Vervoort et al., 2018). Using a structured record form to ensure consistency of reporting, the relationship of the hysteroscopic features with symptoms such as abnormal bleeding, pain, or subfertility can be studied. This relationship can be important if therapeutic options are considered.

Recently there has been an increase in minimally invasive therapies for niche-related symptoms. Besides hormonal therapy, hysteroscopic resection and laparoscopic, vaginal, or abdominal repair are offered. Reviews show that the operative treatment can effectively reduce abnormal uterine bleeding and pain, but 10-36% of women do not benefit from surgical niche treatment (Mashiach and Burke, 2021; Raimondo et al., 2015; Vervoort et al., 2018). Indications and the ideal route for niche repair are still being researched. The selection between hysteroscopic or laparoscopic repair is nowadays made on the thickness of the residual myometrium measured with ultrasound. If the residual myometrium is less than 2.5-3 mm, a laparoscopic repair is usually advised (Mashiach and Burke, 2021; Setubal et al., 2018; Di Spiezio Sardo et al., 2018; Setubal et al., 2021). The clinical relevance of niche features that can be investigated by hysteroscopic evaluation has to be established. It is likely that some features may influence the treatment options and its effect. The presence of cystic formations, crypts or increased vascularity could possibly favour hormonal therapy because adenomyosis may be involved as well (Fernandez et al., 2007; Molinas and Campo, 2006). Patients with accumulated blood, mucus, or dynamic valve in the niche at hysteroscopy may potentially have more benefit from surgical treatment on postmenstrual complaints then those patients without these features. Hysteroscopic biopsy can be obtained to study the relationship between the hysteroscopic features and histology to diagnose the tissue in the niche.

Using a structured record form, a relationship between different items and the effect of therapy can be studied. The use of a standardised hysteroscopic evaluation is essential to be able to compare study outcomes. A standardised hysteroscopic terminology not only helps define the presence or absence of a niche, but actually provides a classification of different niches. Differences in classification can be compared with symptom patterns and the effectiveness of treatment. Additionally, follow-up after structured evaluation could also give more information on the relationship of hysteroscopic features with fertility and pregnancy outcomes. Altogether, uniform hysteroscopic registration when performing a hysteroscopy in patients with CSDi is essential for future research and can potentially contribute to the development of future prognostic models.

## Conclusion

With a modified Delphi method involving international experts in hysteroscopy and ultrasound, a consensus was achieved on the hysteroscopic evaluation and classification of the uterine niche. A standardised record form was developed to support implementation of these recommendations and consistency in reporting. Further studies are needed to validate the classification and to study the relationship between hysteroscopic niche features and symptoms.
