# Supplementary material for: Structured hysteroscopic examination of uterine niches: a modified Delphi procedure
Source: Facts Views Vis Obgyn. 2024 Sep 30;16(3):253–62. doi: 10.52054/FVVO.16.3.036 (PMC11569429; doi:10.52054/FVVO.16.3.036)
Supplement: Supplementary file 1 [file FVVinObGyn-16-253-a001.pdf]

Appendix 1. Questionnaire hysteroscopic registration form uterine caesarean scar

1 Do you think we need a registration form to evaluate niches with hysteroscopy?

A yes, for clinical use

B yes, for research

C yes, for both research and clinical use

D No

2 Which definition do you use to diagnose a niche with hysteroscopy?

A any indentation or disruption of the integrity of the myometrium

B any defect of anterior wall uterus

C Any defect of the anterior wall of the uterus of at least 2 mm

D other:...

3 Should a hysteroscopic evaluation of a niche also include an ultrasound ?

A no

B yes a trans vaginal sonography

C yes a Sonohysterography

Could you explain why ?

....

....

....

Which of the following items are relevant to be included in the registration form?

If you have any comment please feel free to enter it on the line below the question

Position of the niche in relation to the internal ostium of the cervix yes/no

....

Length of the niche yes/no

....

Width of the niche yes/no

....

Depth of the niche yes/no

....

Presence of lateral branches yes/no

....

Presence of cystic formations(including ovula of Nabothi) yes/no

....

Presence of polyp like structures yes/no

....

Presence of crypts yes/no

....

Presence of vessels (amount, small/large/pattern) yes/no

....

Presence of blood yes/no

....

Presence of mucus yes/no

....

Presence of fibrotic tissue yes/no

....

Presence of dynamic valve in the niche (dynamic obstruction of the niche) yes/no

....

5

We are planning to perform an interobserver study to evaluate this registration form.

Her for we will ask you to evaluate 10 videos of hysteroscopic niches online by use of the registration form. Would you like to participate in this interobserver study?

If you will participate, please enter your name and email address below.

Name...

e-mail...

Attached is a example registration form.

Could you please have a look at the form and comment the used definitions and terms ? Are they useful? Is it clear ? Are they possible to identify?

Thank you for willingness to participate and for answering this questionnaire.

The results will be sent to you by email as will the invitations for further research participation.

On behalf of the taskforce on niches

Judith Huirne

Lucet van der Voet
