# Supplementary material for: Structured hysteroscopic examination of uterine niches: a modified Delphi procedure
Source: Facts Views Vis Obgyn. 2024 Sep 30;16(3):253–62. doi: 10.52054/FVVO.16.3.036 (PMC11569429; doi:10.52054/FVVO.16.3.036)
Supplement: Supplementary file 2 [file FVVinObGyn-16-253-a002.pdf]

Appendix 2. Questionnaire second round

Thank you for participating in the DELPHI on hysteroscopic registration of niches.

Introduction

Before we start we would like to inform you on what has been done so far

- *A consensus meeting among Dutch hysteroscopic experts (July 2016) : We consented on:*
  - *The need for a hysteroscopic registration form for niche registration*
  - *A list of potential items to describe.*
- *A first Delphi meeting on hysteroscopic evaluation of the niche taskforce of the ESGE at the ESGE congress in Brussels (October 2016). We consented on:*
  - *Definition of a niche*
  - *The need for a uniform way of registration of a hysteroscopic niche which is additional to the ultrasound registration.*
  - *a list of potential items for registration*

Since not everyone of the expert group was able to join the former meeting we give everyone the opportunity to respond to the consensus of round 1. Additional we will ask some additional questions for further exploration concerning:

- The definition and relevance of the different items to be included in the registration form
- Method of performing a hysteroscopy to identify a niche
- Topics for future research
- To define indications and contra-indications for (hysteroscopic) surgery

We expect to need one or two additional questionnaire rounds by email to achieve full consensus on these items. Consensus is defined as 75% agreement with a response rate of more than 70

%. When no complete consensus can be reach in this two additional e-mail rounds we will organize an expert meeting in June 2017 at the meeting of the niche task force. After consensus has been achieved we will test the interobserver agreement using the registration form among experts and non-experts. It is our aim to develop a final registration form for hysteroscopic evaluation and to write an article on the results of this Delphi procedure, only participants who completed all rounds will be included as a co-author.

Part one confirmation of achieved consensus at the Brussels meeting.

Question 1

> *89% agreed during the previous expert meetings that it is relevant to have a standardized registration form for the evaluation of niches assessed during hysteroscopy for both clinical and research purposes*

Do you agree ?
A Yes
B No, if not please motivate...

....

Question 2:

> ***definition***
> *89% agreed with the following definition of a niche evaluated during hysteroscopy: any indentation in the myometrium at the site of a previous CS*

Do you agree with this definition?

A Yes
B No, if not please motivate

What definition do you propose in case of B:……………………….

…………………………………………………………………………….

Question 3

> *There was complete consensus (100%) that a hysteroscopic evaluation of a niche has to be combined with an ultrasound evaluation in order to measure the residual myometrium.*

Do you agree?
A yes
B No, if not please motivate

Question 4

For the development of a registration form there was consensus for the following items to be included

- *Presence of lateral branches (Consensus 89%)*
- *Presence of cystic formations (including ovula of Nabothi) (Consensus 78%)*
- *Presence of polyp like structures (Consensus 100%)*
- *Presence of crypts (Consensus 78%)*
- *Presence of vessels (amount, small/large/pattern) (Consensus 100%)*
- *Presence of blood (Consensus 100%)*
- *Presence of mucus (Consensus 89%)*
- *Presence of fibrotic tissue (Consensus 78%)*
- *Presence of dynamic valve in the niche (dynamic obstruction of the niche) (Consensus 100%)*

Could you please answer for every item separate if you agree to include this item in the registration form

Do you agree with all items:

A if yes, please continue with question 5

B if no please complete the following questions:

4a Do you agree to include presence of lateral branches in the registration form?

A yes

B No, please motivate

4b Do you agree to include presence of cystic formations (including ovula of Nabothi) in the registration form?

A yes

B No, please motivate

4c Do you agree to include presence of polyp like structures in the registration form?

A yes

B No, please motivate

4d Do you agree to include presence of crypts in the registration form?

A yes

B No, please motivate

4e Do you agree to include presence of vessels (amount, small/large/pattern) in the registration form?

A yes

B No, please motivate

4f Do you agree to include presence of blood in the registration form?

A yes

B No, please motivate

4g Do you agree to include presence of mucus in the registration form?

A yes

B No, please motivate

4h Do you agree to include presence of fibrotic tissue in the registration form?

A yes

B No, please motivate

4i Do you agree to include presence of dynamic valve in the niche (dynamic obstruction of the niche) in the registration form?

A yes

B No, please motivate

Question 5

During the expert meeting there was consensus (100.%)that measuring the depth and of the niche in mm or cm is not useful since it can't be done accurately during hysteroscopy. This has to be measured with ultrasound. However it was consensus (100%) that a subjective qualification of the seize or volume of the niche is useful.

5a Do you agree that measuring the depth and width in a quantitative way (in mm or cm) should be measured during ultrasound

A Yes

B No because...

If you answered No how will you propose to measure the depth and width of the niche during hysteroscopy

....

5b Do you agree that registration of a size indication/estimation of the niche (for example in relation to the size of the uterine cavity or to the size of the width of the endocervix) could be useful?

A Yes

No because

Part two Achieving consensus

On the following potential items there was no consensus yet during the meeting or it has not been discussed yet.

There was consensus to describe the size of the uterus in a subjective way. Some suggestions have been proposed to describe the size in a subjective manner.

Question 6

How would you prefer to describe the size of the niche

A In relation to the size of the uterine cavity (for example smaller than, as big as, or bigger than)

B In relation to the width of the cervical canal (for example the size is less than 50% of the with of the cervical canal or more than 50% - 100% or > 100%)

C other, please give your definition……………………………………………………..

……………………………………………………………………………………………….

Please motivate your given answer………………………………………..

50% found it relevant to describe where the niche was located in relation to the internal os
50% thinks this is not useful since a niche itself can form a new internal os depending on the location of the niche.

In the achieved consensus on niche measurements with ultrasound the position in relation to the internal os was not included in niche measurement for the same reason as mentioned above

Question 7

Do you agree that it is NOT relevant to register the position of the niche in relation to the internal os in line with the achieved consensus for niche measurement using ultrasound?

A Yes, please motivate...

B No, please motivate...

Question 8

How would you define the location of the niche in the uterus:

A As … mm distance of the external os

B Others, ……..

Question 9

Do you think it is relevant to add a subjective judgement if you think the niche is in the cervical canal or in the uterine cavity

A Yes, please motivate……

B No, please motivate……,

The following items were new suggested during the Brussels meeting to be added to the registration form

The presence of endometrium in the niche

The presence of placental tissue

Question 10 a

Do you agree it is relevant to register the presence of endometrium in the niche

A yes, please motivate

B No , please motivate………

Question 10b

Do you agree it is relevant to register the presence of placental tissue

A Yes, please motivate………………

B No, please motivate………………………..

We tried to define all possible items that were found to be relevant on the meeting.

We will show you some pictures and recordings of various items and we will ask you how to define them

Question 11

Polyps

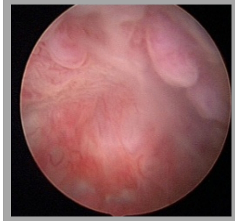

o[illegible]

A a pedunculated structure focally growing marginate mass with soft texture

B a focally growing margined mass with a soft texture

C a focally growing margined mass with an endometrial like texture

D Other, please motivate

Could you motivate your answer

Question 12

Branches

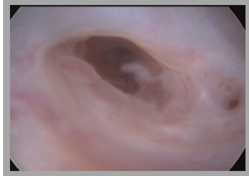

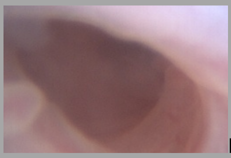

recording 6 / 7

During the ultrasound measurement Delphi there was consensus achieved on the following definition of a branch
A smaller part of the niche directing towards the serosa that has a smaller width then the niche itself.

Do you agree with this definition

A yes, please motivate

B No, please motivate

Question 13

Cystic formations

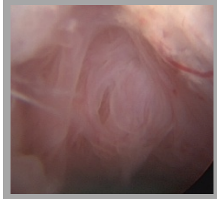 recording 8

Including ovula Nabothi and inclusion cysts

Do you agree with the following definition:

round secluded cavity filled with fluid visible by bulging of the surface of the niche

A yes Please motivate

B No Please motivate

Question 14

Recording 9 9 crypts

Definition of crypts

Crypts are defined as cysts with an open connection to the niche

Do you agree with this definition

A yes, please motivate

B No, Please motivate

Question 15

Vessels

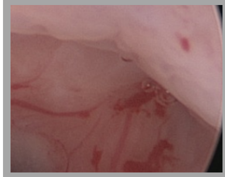

You can define different aspects of vessels like , amount, pattern
size (small/large), bleeding when releasing pressure.
Do you think it is relevant to describe these items?

What aspects of vessels in the niche need to be included in the registration form? (more options are allowed)

15a amount of vessels

A yes, please motivate

B No, Please motivate

15b pattern of vessels

A yes, please motivate

B No, Please motivate

15c the size of the vessels (large/small)

A yes, please motivate

B No, Please motivate

15d Easily bleeding vessels (after lowering infusion pressure)A yes, please motivate

B No, Please motivate

15e other, please specify

Question 16

How would you define fibrotic tissue

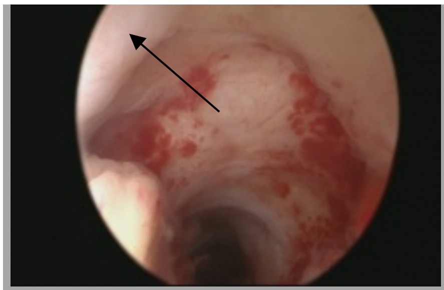

fibrotic tissue

We define fibrotic tissue as a focal hard tissue with a white surface without vessels

Do you agree

A yes, continue with question 17

B No, Please complete following questions

16a White tissue

A yes, please motivate

B No, Please motivate

16b Absence of vessels

A yes, please motivate

B No, Please motivate

16c hard tissue

A yes, please motivate

B No, Please motivate

16d Other definition: ………………… , please motivate…………………

Question 17

recording dynamic valve niche

We defined the presence of dynamic valve in the niche as

A a dynamic partial or complete obstruction of the niche outflow which moves when changing pressure

Do you agree with this definition?

A yes, please motivate

B No, Please motivate

The following items have not been discussed in Brussels and will be discussed in this Delphi

Question 18

18a Do you think it is relevant to describe the amount of niches

A yes, please motivate

B No, Please motivate

18b Which niche should be described when there are more than one niches?

the largest

A yes, please motivate

B No, Please motivate

18c The lowest (closest towards the external os of the cervix)

A yes, please motivate

B No, Please motivate

Question 19

Using sonography we consented to classify niches as simple, simple with one branch and complex

Do you think we should use the same classification during hysteroscopy?

A yes, please motivate

B No, Please motivate

Part Three Methods of hysteroscopic evaluation in the evaluation of niches:

Question 20

In order to increase the inter-observer agreement it is relevant to standardise the hysteroscopic niche evaluation we proposed:

To identify and evaluate a niche in a diagnostic setting a diagnostic hysteroscopy is performed without dilatation.

The diameter of the diagnostic hysteroscope influences the outcome. Therefore the diameter of the scope should be registered.

Question 20 a

Do you agree that it is most ideal to identify and evaluate a niche in a diagnostic setting a diagnostic hysteroscopy without dilatation, since a niche can change after dilatation?

A Yes, please motivate

B No, please motivate

Question 20 b

Since dilatation may change niche appearance, it seems to be logical to register the diameter of the hysteroscope during evaluation.

Do you agree?

A: yes, please motivate

B: no, please motivate.

Question 21

Do you think it is preferable to capture the niche in pictures or in film as part of a registration form?

A yes

B No

Please motivate

Question 22

If you want to capture the niche in pictures or film the following aspects has to be included

Please answer for every item if you agree

22a overview of cervical canal and niche

A Yes, please motivate

B No, please motivate

22 b separate pictures of the left and right corner of the niche

A Yes, please motivate

B No, please motivate

22 c separate pictures of lateral branches, polyp like structures and other abnormalities in the niche

A Yes, please motivate

B No, please motivate

22d Picture/movie of uterine cavity with tuba ostia

A Yes, please motivate

B No, please motivate.

22e With and without pressure to show if vessels will bleed

A Yes, please motivate

B No, please motivate
