# Supplementary material for: Structured hysteroscopic examination of uterine niches: a modified Delphi procedure
Source: Facts Views Vis Obgyn. 2024 Sep 30;16(3):253–62. doi: 10.52054/FVVO.16.3.036 (PMC11569429; doi:10.52054/FVVO.16.3.036)
Supplement: Supplementary file 3 [file FVVinObGyn-16-253-a003.pdf]

Appendix 3. Schematic diagram of niche characteristics during hysteroscopy

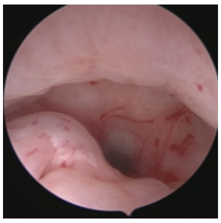
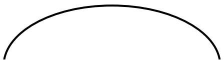

1.concavity in anterior wall

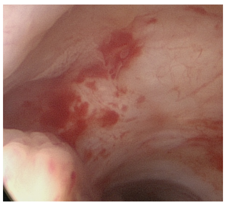
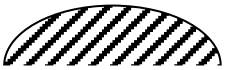

2. mucosa

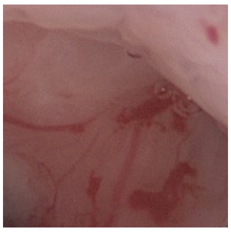
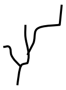

3.abnormal vascular pattern

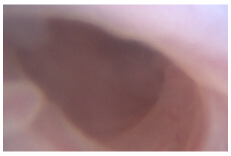
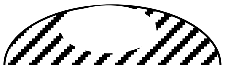

4.serosa visible /defect myometrium

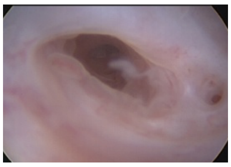
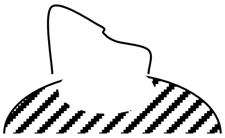

5.lateral branches

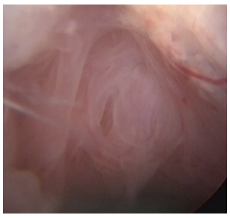
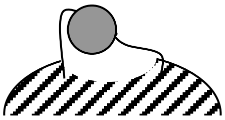

6.cystic formations

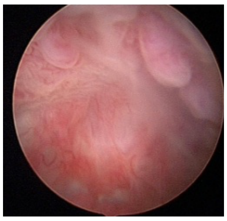
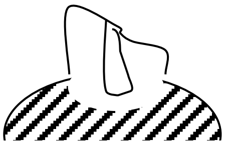

7.polyp like structures
